# Diminished Prognostic Value of Interim PET in Hodgkin Lymphoma Treated with Brentuximab Vedotin Plus AVD: A Retrospective Analysis

**DOI:** 10.3390/hematolrep18040053

**Published:** 2026-07-31

**Authors:** Kareem Latif, Isaiah Courtad, Avery Stuart, Faisal Ansari, Ravand Samaeekia, Mojtaba Akhtari

**Affiliations:** 1 Department of Medicine, Loma Linda University School of Medicine, Loma Linda University Medical Center, Loma Linda, CA 92350, USA; kareemlatif@llu.edu (K.L.); fansari@llu.edu (F.A.); 2 School of Medicine, Loma Linda University School of Medicine, Loma Linda, CA 92350, USA; icourtad@students.llu.edu (I.C.); astuart@llu.edu (A.S.); 3 Department of Medicine, Division of Hematology/Oncology, University of California Irvine, Irvine, CA 92868, USA; rsamaeek@hs.uci.edu; 4 Department of Medicine, Division of Transplant, Cellular Therapy, and Hematological Malignancies, Loma Linda University School of Medicine, Loma Linda University Medical Center, Loma Linda, CA 92350, USA

**Keywords:** Hodgkin lymphoma, brentuximab vedotin, positron emission tomography, response-adapted therapy

## Abstract

**Background**: Interim PET after two cycles (PET2) has historically guided risk-adapted therapy in Hodgkin lymphoma (HL). Its value with frontline brentuximab vedotin plus doxorubicin, vinblastine, and dacarbazine (BV + AVD) is uncertain, as BV + AVD improves progression-free survival and may reduce the prognostic relevance of early metabolic response. Our aim was to assess whether PET2 retains prognostic utility in HL patients treated with BV + AVD. **Methods**: We retrospectively reviewed 55 newly diagnosed classical HL patients treated with frontline BV + AVD. The primary endpoint was PET2 performance for predicting EOT PET positivity. Sensitivity, specificity, predictive values, and likelihood ratios were calculated with 95% confidence intervals. **Results**: Median age was 22 years (range 11–77), 49% were male, and 73% had stage III–IV disease. PET2 was negative in 51 patients (92.7%) and positive in 4 (7.3%). EOT PET was negative in 48 (87.3%) and positive in 7 (12.7%). Of seven EOT PET-positive patients, three were PET2-positive and four PET2-negative. Of 48 EOT PET-negative patients, 47 were PET2-negative and 1 PET2-positive. PET2 sensitivity was 42.9%, specificity 97.9%, positive predictive value 75.0%, and negative predictive value 92.2%. Positive likelihood ratio was 20.6; negative likelihood ratio was 0.58. **Conclusions**: In HL patients treated with BV + AVD, most achieve PET2 negativity, limiting interim PET discrimination. Negative PET2 adds little reassurance beyond the high baseline success rate, while positive PET2 is uncommon and identifies only a subset of eventual treatment failures. These findings question the routine use of PET2 to guide therapy in the BV + AVD era.

## 1. Introduction

PET after two cycles of chemotherapy (PET2) has become standard practice in HL due to its strong prognostic value in the pre-brentuximab era [1]. Seminal studies demonstrated that an early metabolic response is highly predictive of outcome. For example, Gallamini et al. reported that PET2 results “overshadow” the International Prognostic Score (IPS) as a risk factor, emerging as the single most important tool for risk-adapted therapy in advanced HL [2]. In patients treated with conventional ABVD (doxorubicin, bleomycin, vinblastine, dacarbazine), a negative PET2 is associated with long-term remission rates around 90–95%, whereas a positive PET2 portends a very high risk of treatment failure [2]. This enabled PET2-guided strategies: trials like RATHL (Response-Adapted Therapy in HL) adopted PET2 to escalate therapy for high-risk patients. In the RATHL study, approximately 16% of advanced-stage patients had positive interim PET and were switched to more intensive BEACOPP (bleomycin, etoposide, adriamycin, cyclophosphamide, oncovin, procarbazine, and prednisone) chemotherapy, achieving a 3-year PFS of 67.5%, which is inferior to the 85.7% PFS of PET2-negative patients who continued ABVD [3]. These findings solidified interim PET as a critical decision point in managing HL.

The therapeutic landscape of HL changed with the introduction of brentuximab vedotin, an anti-CD30 antibody-drug conjugate. The phase 3 ECHELON-1 trial demonstrated that adding brentuximab vedotin to AVD (BV + AVD, replacing bleomycin) led to superior efficacy compared to ABVD in stage III–IV HL [4]. After a median follow-up of about 3–6 years, BV + AVD has shown improved progression-free and overall survival (OS) in advanced HL, and it has been adopted as a frontline standard for many patients [5]. With more effective frontline regimens, it is conceivable that early PET response might be less prognostic than before, as even patients with a slower early response could still be salvaged or cured by the end of therapy. Indeed, emerging data suggest that the prognostic contribution of PET2 may be blunted in the context of BV-containing therapy. A recent meta-analysis of frontline trials found that while BV-based regimens trend toward higher PET2-negativity rates, the difference in interim metabolic response between BV + AVD and traditional regimens was not statistically significant [5]. Moreover, BV-based therapy yielded significantly higher complete metabolic response rates at end-of-treatment, implying that final outcomes improved regardless of interim PET status.

Several BV-era studies have raised uncertainty regarding the prognostic role of interim PET. In ECHELON-1, BV + AVD was delivered without PET-adapted treatment modification and demonstrated durable PFS and OS benefit compared with ABVD, with benefit observed regardless of PET2 status [6,7]. Similarly, the BREACH trial demonstrated a higher PET2-negative rate with BV + AVD compared with ABVD after two cycles and excellent 2-year PFS in early unfavorable HL [8]. However, emerging real-world data suggest that PET2 may retain prognostic significance in selected high-risk populations; for example, Rusconi et al. reported inferior 1-year PFS among PET2-positive stage IV HL patients treated with frontline BV + AVD [9]. Together, these findings suggest that interim PET may have reduced discriminatory value in the BV era overall, but its role in specific high-risk subgroups remains uncertain.

In this context, we conducted a retrospective study to examine the predictive performance of interim PET in a cohort of patients with classical HL treated with frontline BV + AVD. We aimed to quantify the sensitivity, specificity, and predictive values of PET2 for end-of-treatment disease status. We hypothesized that due to the high efficacy of BV + AVD, the prognostic value of PET2 would be diminished compared to historical ABVD-treated cohorts. Our findings shed light on the utility of interim PET in the BV + AVD era and inform whether risk-adapted strategies are still warranted with this regimen.

## 2. Materials and Methods

### 2.1. Study Design and Patients

We performed a retrospective chart review of patients with classical Hodgkin lymphoma treated at our institution with frontline BV + AVD between 2016 and 2024. Inclusion required newly diagnosed, biopsy-proven classical HL and intention to treat with curative frontline BV + AVD. We identified 112 consecutive patients with newly diagnosed classical HL treated with frontline BV + AVD during the study period. Of these, 57 patients were excluded because PET2 and/or EOT PET imaging data were unavailable. The final analytic cohort included 55 patients who had both PET2 and EOT PET available for review. This cohort spanned pediatric, adolescent, and adult patients (age range 11–77). Clinical data were obtained from electronic medical records under an IRB-approved protocol.

### 2.2. Treatment and Assessments

All patients received BV + AVD chemotherapy, consisting of brentuximab vedotin (1.2 mg/kg) plus AVD given in 28-day cycles. Most patients with advanced stage (III–IV) received six cycles (approximately 12 doses of BV), while early-stage patients (stage II, often with bulky disease or other risk factors) typically received 4–6 cycles. Interim restaging with PET/CT was performed after two cycles of therapy (PET2), and treatment was continued to the planned 4–6 cycles regardless of PET2 results in nearly all cases. Changes in therapy based on PET2 were not protocolized; any escalation or alteration was at the treating physician’s discretion. At the completion of planned first-line therapy, all patients underwent an EOT PET/CT evaluation to assess remission status. Additional radiologic or biopsy confirmation was obtained as clinically indicated for PET-positive findings.

### 2.3. PET Interpretation

PET scans were interpreted by board-certified nuclear medicine radiologists and reported using the Deauville 5-point scoring system, the standard in HL response assessment. For this study, we defined PET2 “negative” as Deauville score 1–3 (no or low-level uptake, up to mild uptake < mediastinal blood pool or ≤liver background) and PET2 “positive” as Deauville 4–5 (uptake moderately to markedly above liver, or new focal disease). These criteria align with international consensus and Lugano 2014 response guidelines for interim PET in HL [10]. EOT PET was interpreted similarly, with Deauville 1–3 considered complete metabolic response and 4–5 indicating residual active disease. PET results were extracted from the patients’ radiology reports.

### 2.4. Data Collection

Patient demographics, baseline disease characteristics, treatment details, and outcomes were collected. Baseline characteristics included age at diagnosis, sex, ethnicity, Ann Arbor stage, presence of B symptoms, number of extranodal sites, IPS, performance status, and histologic subtype. Key time points (diagnosis date, treatment start and end dates, PET dates, and last follow-up date) were recorded to calculate follow-up duration. PET2 and EOT PET Deauville scores were recorded from reports. The primary outcome of interest was EOT remission status by PET (metabolic complete response vs. residual uptake). We also noted any therapy modifications triggered by PET2 (such as early escalation or switch of regimen) and any post-treatment relapses or additional therapies during follow-up.

### 2.5. Statistical Analysis

We constructed a 2 × 2 contingency table of interim PET2 results versus end-of-treatment PET results. From this, we calculated the sensitivity, specificity, positive predictive value (PPV), negative predictive value (NPV), and positive/negative likelihood ratios (LR+ and LR–) of PET2 for predicting a positive EOT PET. Sensitivity was defined as the proportion of end-of-treatment positive cases that had a positive interim PET2. Specificity was the proportion of end-of-treatment negative cases that had a negative PET2. PPV was the probability of end-of-treatment positivity given a positive PET2, and NPV the probability of end-of-treatment negativity given a negative PET2. Exact 95% confidence intervals (CI) for these proportions were calculated using the Wilson score method. In addition, we summarize baseline characteristics using descriptive statistics (median, range, and proportions). The follow-up time was calculated from the date of diagnosis to the date of last follow-up or death. We report the median follow-up in the cohort. No formal PFS or OS analysis stratified by PET2 status was performed because the number of PET2-positive patients and subsequent progression or survival events was limited, making time-to-event comparisons underpowered and potentially unstable. Therefore, the primary analysis focused on the diagnostic performance of PET2 for predicting EOT PET positivity. EOT PET response was used as an early surrogate endpoint rather than a definitive measure of long-term disease control. Relapsed or refractory cases, when available, were summarized descriptively according to PET2 and EOT PET status. All analyses were conducted using R 4.5.1, and a two-tailed *p* < 0.05 was considered significant for any comparisons.

## 3. Results

### 3.1. Patient Characteristics

Fifty-five patients with HL treated with BV + AVD were included in this analysis (Table 1). The median age at diagnosis was 22 years (range 11–77). Stage IV disease was present in 40% of patients, stage III in 33%, and stage II in 27%. The International Prognostic Score (IPS) was ≥3 in 58% of patients, reflecting a high-risk population. B symptoms were present in 66% of patients. At end of treatment, 48 patients (87%) achieved complete remission, consistent with the high efficacy of BV + AVD in this population.

### 3.2. Interim PET2 and End-of-Treatment Results

All 55 patients underwent an interim PET/CT scan after 2 cycles of BV + AVD (PET2). At interim restaging, an overwhelming majority of patients were PET2-negative. Specifically, 51 of 55 patients (92.7%) achieved PET2-negative status, whereas only 4 patients (7.3%) had a positive interim PET (Deauville 4–5). All patients in our series completed their planned therapy regardless of PET2 findings. No patient underwent an interim treatment intensification to BEACOPP.

At the end of therapy, PET/CT was performed in all patients to assess treatment response. Overall, the end-of-treatment PET (EOT PET) results were excellent, reflecting the high efficacy of BV + AVD. A total of 48 patients (87.3%) achieved a negative EOT PET, indicating complete metabolic response and likely complete remission. The remaining 7 patients (12.7%) had a positive EOT PET, indicative of residual active disease or treatment failure. These 7 represent the subset with an inadequate response to initial therapy, either refractory disease or early relapse detected on the post-treatment scan.

The relationship between interim PET2 results and EOT outcomes is shown in Table 2. Of the 7 patients with a positive EOT PET, 3 had been PET2-positive at interim and 4 had been PET2-negative. Conversely, of the 48 patients who achieved EOT PET negativity, 47 had been PET2-negative and 1 patient had been PET2-positive.

In an exploratory review of discordant cases, all 4 patients with false-negative PET2 results, defined as PET2-negative but EOT PET-positive disease, had advanced-stage HL, with stage III–IV disease at diagnosis. Given the small number of discordant events, no formal subgroup analysis by stage, IPS, age group, or B symptoms was performed.

### 3.3. Predictive Performance of PET

**Figure 1:**
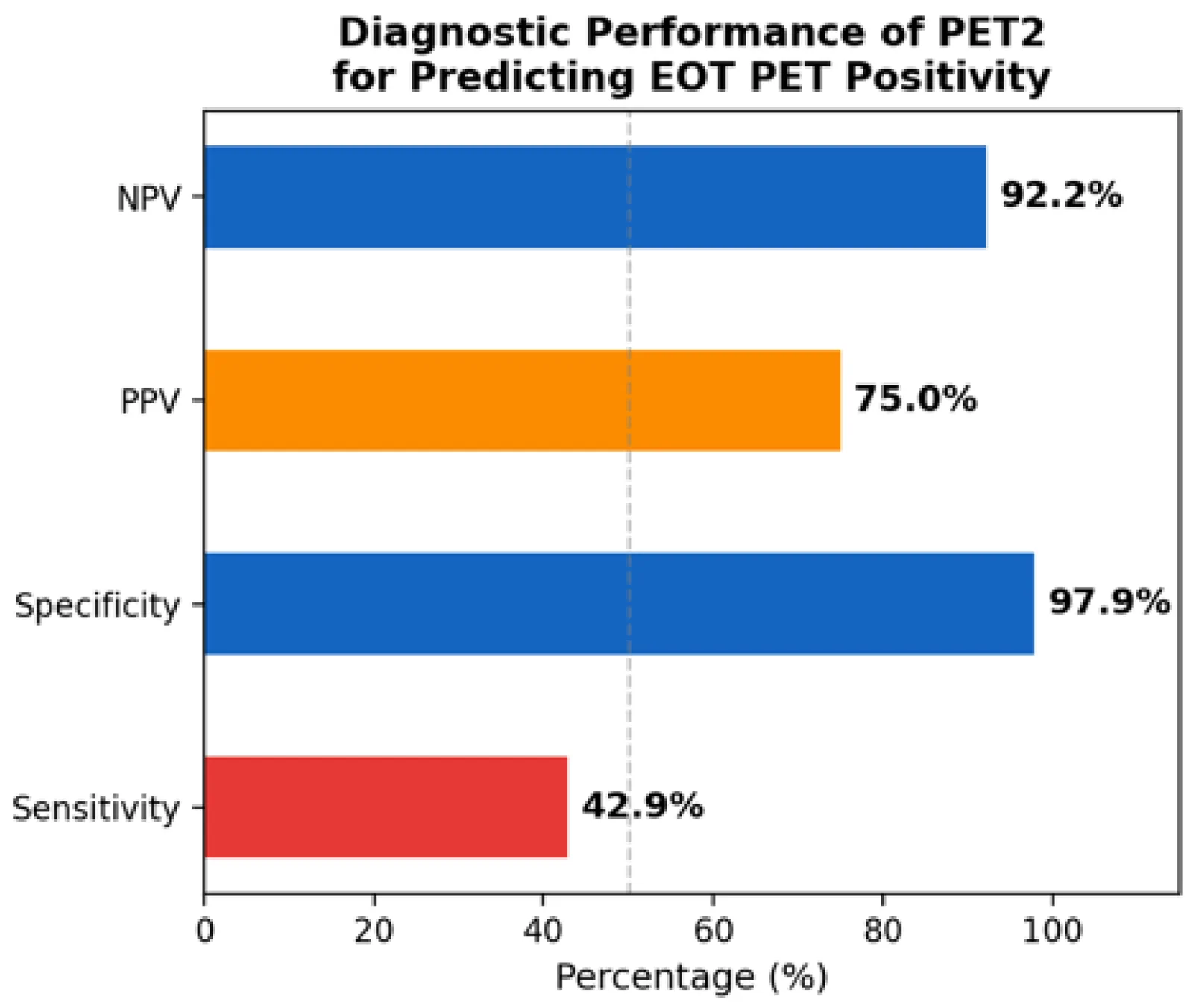
Diagnostic performance of PET2 for predicting EOT PET positivity. PET2 demonstrated high specificity (97.9%) and negative predictive value (92.2%), but limited sensitivity (42.9%), indicating reduced ability to identify patients who will ultimately have persistent disease.

Diagnostic performance metrics with 95% confidence intervals were computed and illustrated in Figure 1. Sensitivity was 42.9% (95% CI 15.8–75.0%), indicating that PET2 correctly identified fewer than half of patients who would ultimately be EOT PET-positive, missing 4 of 7 patients (57.1%) with unfavorable EOT imaging outcomes despite a negative interim scan. The wide confidence interval reflects the small number of EOT-positive events and underscores the imprecision of PET2 as a predictive test in this setting. Specificity was 97.9% (95% CI 89.1–99.6%), reflecting the near-universal PET2 negativity in this cohort. While numerically high, this value is driven by the low prevalence of EOT PET positivity rather than the true discriminatory power of the test.

Positive predictive value (PPV) was 75.0% (95% CI 30.1–95.4%) and negative predictive value (NPV) was 92.2% (95% CI 81.5–96.9%). The NPV, while seemingly reassuring, is heavily inflated by the low pre-test probability of EOT PET positivity (12.7%) and does not reflect meaningful discriminatory ability of PET2. Overall diagnostic accuracy was 90.9% (95% CI 80.4–96.1%), similarly driven by the high proportion of true-negative results.

### 3.4. Likelihood Ratios and Post-Test Probability

To evaluate the clinical utility of PET2 independent of disease prevalence, likelihood ratios were calculated. The positive likelihood ratio (LR+) was 20.57 (95% CI 2.47–171.34), indicating that a positive PET2 result substantially increases the post-test probability of EOT PET positivity, from a pre-test probability of 12.7% to a post-test probability of 75.0% (95% CI 26.5–96.2%). However, this applies to only 4 patients (7.3%) in the cohort.

Critically, the negative likelihood ratio (LR−) was 0.584 (95% CI 0.307–1.110). This value is far above the threshold of ≤0.10 generally required for a test to be considered clinically useful for ruling out an outcome. Accordingly, a negative PET2 result reduced the post-test probability of EOT PET positivity only marginally, from 12.7% to 7.8% (95% CI 4.3–13.9%), a clinically negligible shift that provides minimal incremental reassurance beyond the pre-test probability alone. Notably, the upper bound of the 95% CI for LR− crosses 1.0, meaning that in the least favorable scenario, a negative PET2 result does not reduce the probability of EOT PET positivity at all.

**Figure 2:**
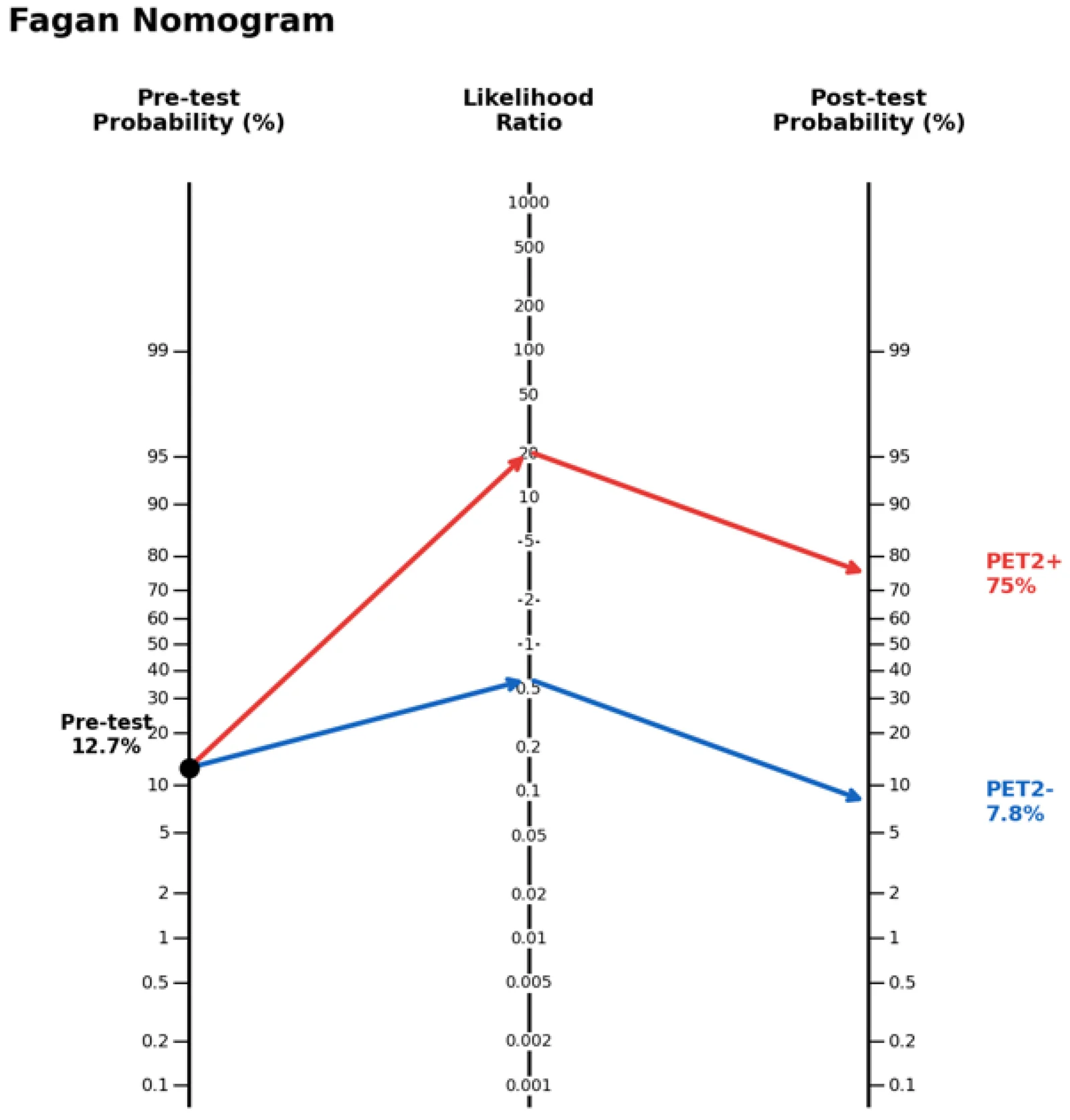
Fagan nomogram for PET2 and EOT PET outcome. Using a pre-test probability of 12.7%, a positive PET2 increased the post-test probability of EOT PET positivity to 75.0%, whereas a negative PET2 reduced it only to 7.8%, demonstrating limited rule-out utility.

Using the observed EOT PET positivity rate of 12.7% as the pre-test probability, a Fagan nomogram (Figure 2) was constructed to assess the clinical impact of PET2 on post-test risk estimation. A positive PET2 result, corresponding to an LR+ of 20.57 (95% CI 2.47–171.34), increased the post-test probability of EOT PET positivity from 12.7% to 75.0% (95% CI 26.5–96.2%). In contrast, a negative PET2 result, corresponding to an LR− of 0.584 (95% CI 0.307–1.110), reduced the post-test probability only marginally, from 12.7% to 7.8% (95% CI 4.3–13.9%). Visually, the nomogram demonstrates that the positive test trajectory produces a substantial upward shift in probability, whereas the negative test trajectory produces only a small downward shift. This finding reinforces the limited rule-out value of PET2 in patients treated with BV + AVD, as a negative interim PET does not meaningfully decrease the probability of an unfavorable EOT PET outcome beyond the baseline pre-test risk.

## 4. Discussion

In this retrospective analysis of 55 patients with classical HL treated with frontline BV + AVD, we found that the predictive value of interim PET2 was markedly diminished compared to historical experience with ABVD. Only 7% of our cohort had a positive interim PET after two cycles, which is a low incidence relative to prior studies in the pre-BV era [3]. This finding alone suggests that BV + AVD induces metabolic remissions in the vast majority of patients early in therapy. The addition of brentuximab vedotin likely debulks disease more rapidly or effectively, so that very few patients demonstrate inadequate response at interim restaging. Consistent with this, nearly 93% of our patients were PET2-negative. This high early response rate aligns with the known efficacy of BV + AVD [8]. The ECHELON-1 trial did not incorporate risk-adaptive therapy because outcomes were expected to be uniformly improved in the experimental arm [7]. Our real-world data confirm that an interim scan is usually negative when using this regimen.

Crucially, our results show that a negative PET2 is less informative under these circumstances. With ABVD, a negative PET2 was a strong predictor of durable remission (NPV ~95%, and patients with negative interim scans had excellent outcomes unless treatment was reduced too far). In our BV + AVD cohort, the NPV of PET2 was 92%, which on the surface appears similarly high. However, one must consider that the baseline likelihood of achieving remission with BV + AVD was already ~87% in our study. Thus, PET2 negativity only modestly improved the estimated chance of cure. In practical terms, a patient who is PET2-negative on BV + AVD is almost certainly responding, but their ultimate prognosis was already very favorable with continued therapy. The negative interim scan does not substantially change management or provide much additional reassurance beyond what we expect from the regimen itself.

Comparing our findings to the literature, historically, interim PET has been one of the most powerful predictors of outcome in HL, guiding risk-adapted therapy for over a decade [11]. In the pre-BV era, trials demonstrated that patients with persistent uptake after two cycles of ABVD had a very poor prognosis with continued standard therapy, but could achieve improved disease control if treatment was escalated [12]. This formed the basis of trials like RATHL and EORTC/UK NCRI RAPID, establishing PET-driven escalation and de-escalation as standard of care [3,13,14]. However, our findings align with several key studies suggesting that the landscape has shifted with the advent of BV.

The ECHELON-1 trial’s results hinted at this shift; despite no PET-adaptation, A + AVD led to significantly better modified PFS than ABVD across all subgroups [4]. Subsequent subgroup analysis showed A + AVD conferred an OS benefit both for PET2-negative patients and for PET2-positive patients [7]. In fact, PET2-positive patients fared remarkably well on A + AVD, with a 6-year OS of ~95%, far higher than historical outcomes for interim PET-positive disease. Additionally, investigators reported that very few patients on the BV arm required regimen change during therapy. This implies that continuing BV + AVD through six cycles was effective for most patients, even those with suboptimal early response. The BREACH trial similarly found that BV + AVD improved PET2-negative rates and PFS relative to ABVD [8]. Notably, in BREACH, 82% of patients on BV + AVD became PET-negative after two cycles, versus 75% on ABVD. More importantly, 2-year PFS in the BV arm was 97%, indicating that even some PET2-positive patients must have been salvaged by ongoing therapy or subsequent treatment, because the PFS was far higher than the PET2-negative rate. Although BREACH was not explicitly PET-adaptive, it reinforces the theme that BV-based therapy mitigates the risk of early PET positivity.

The findings of the BREACH trial, together with those from the ECHELON-1 trial, provide important context for interpreting our results. Both studies demonstrate that BV-containing regimens produce high early response rates and favorable PFS irrespective of interim PET status, suggesting that early metabolic response has diminished prognostic value in this setting. In BREACH, the 2-year PFS of 97% exceeded the PET2-negative rate, indicating that a proportion of PET2-positive patients achieved durable remission with continued therapy. Similarly, ECHELON-1 showed improved outcomes with BV + AVD across subgroups, without clear dependence on interim PET response. In our cohort, this paradigm is reflected by the low pre-test probability of treatment failure and the limited ability of PET2 to meaningfully alter post-test risk, despite a measurable proportion of PET2-positive patients. Consistent with these trials, we observed that PET2 status did not reliably stratify outcomes, reinforcing that the efficacy of BV + AVD mitigates the prognostic significance of interim PET.

This study has important limitations that warrant careful interpretation. First, the cohort was modest in size and derived from a single institution, with only 55 patients included in the final analysis. More importantly, the number of informative events was small: only four patients were PET2-positive and only seven patients were EOT PET-positive. As a result, several diagnostic performance estimates, including sensitivity, PPV, and LR+, were associated with wide 95% confidence intervals, limiting precision. Therefore, although the point estimates suggest diminished rule-out utility of PET2 in patients treated with BV + AVD, the true performance of interim PET could differ in a larger and more heterogeneous population. These findings should be interpreted as hypothesis-generating rather than definitive. Second, PET scans were not centrally reviewed. PET2 and EOT PET results were extracted from clinical radiology reports interpreted by local board-certified nuclear medicine radiologists rather than adjudicated by a blinded central review panel. Although this reflects real-world practice, it introduces potential inter-observer variability in Deauville scoring, particularly for borderline Deauville 3 versus 4 cases, which could affect PET-positive versus PET-negative classification and influence diagnostic performance estimates. Third, although the median follow-up was 38 months, late relapses in Hodgkin lymphoma can occur beyond this timeframe. In addition, the small number of PET2-positive patients and limited number of progression or survival events precluded meaningful PFS or OS analyses stratified by PET2 status. Therefore, our study relied on EOT PET response as an early surrogate endpoint rather than long-term disease control. While EOT PET positivity is clinically meaningful, it should not be interpreted as uniformly biopsy-proven refractory disease. Confirmatory biopsy, subsequent relapse/progression, and post-EOT treatment data were not uniformly available, and inflammatory or treatment-related uptake may lead to false-positive PET findings. Thus, use of EOT PET positivity as the primary endpoint may introduce misclassification and limit conclusions about long-term outcomes.

Finally, the cohort included pediatric, adolescent, and adult patients, with an age range of 11–77 years, as well as both early- and advanced-stage disease. This introduces potential biological and treatment heterogeneity, as disease biology, treatment tolerance, supportive care practices, and relapse patterns may differ across age groups. Because of the small number of PET2-positive and EOT PET-positive events, formal subgroup analyses by age, stage, IPS, bulky disease, or B symptoms were not feasible. Notably, all four PET2 false-negative cases occurred in patients with stage III–IV disease, supporting the need for larger studies to determine whether PET2 retains prognostic value in advanced-stage or otherwise high-risk subgroups. Larger prospective, multi-center studies with standardized PET interpretation, central review, biopsy confirmation when feasible, treatment data, mature PFS/OS follow-up, and age-specific analyses are needed to validate these findings and better define the clinical significance of PET2 and EOT PET positivity.

## 5. Conclusions

In this single-center retrospective cohort of patients with classical Hodgkin lymphoma treated with frontline BV + AVD, PET2 after two cycles was negative in more than 90% of patients and had limited ability to identify the small subset with EOT PET positivity. However, these findings should be interpreted cautiously given the modest sample size, very small number of PET2-positive and EOT PET-positive cases, wide confidence intervals, lack of central PET review, reliance on EOT PET positivity as an imaging surrogate, and absence of PET2-stratified PFS or OS analyses. Therefore, our results should be considered hypothesis-generating rather than practice-changing. They suggest that the prognostic role of interim PET in BV + AVD-treated HL may be less discriminatory than in historical ABVD-treated cohorts, but they do not establish that PET2 can be safely omitted or de-emphasized in routine clinical practice. Larger prospective, multi-center studies with standardized PET interpretation, central review, biopsy confirmation of PET-positive findings when feasible, and mature PFS and OS follow-up are needed to determine whether PET2 retains prognostic value after frontline BV + AVD and whether any high-risk subgroups may still benefit from interim PET-guided treatment adaptation.

## Figures and Tables

**Table 1:** Patient demographics and baseline disease characteristics (*N* = 55).

Characteristic	
Total patients	55
Male sex—no. (%)	24 (43%)
Age (yr)	
Median (range)	22 (11–77)
Age categories—no. (%)	
<21	26 (46%)
22–45	23 (42%)
>45	7 (12%)
Ethnicity—no. (%)	
Hispanic	23 (42%)
Non-Hispanic White	23 (42%)
Non-Hispanic Black	9 (15%)
Ann Arbor stage at initial diagnosis—no. (%)	
Stage II	15 (27%)
Stage III	18 (33%)
Stage IV	22 (40%)
International Prognostic Score—no. (%)	
Low (0–2)	23 (42%)
High (3–7)	32 (58%)
ECOG performance status—no. (%)	
0	27 (48%)
1	14 (25%)
2	13 (23%)
3	2 (4.0%)
Extranodal involvement at diagnosis—no. (%)	
Yes	36 (64%)
1 extranodal site	23 (41%)
>1 extranodal site	13 (23%)
No	20 (36%)
Patients with any B symptom—no. (%)	37 (66%)
Median follow-up in months	38
Death—no./total no. (%)	1/55 (2.0%)
Deauville score at PET2—no. (%)	
0–3 (PET negative)	51 (93%)
>3 (PET positive)	4 (7.3%)
Deauville score at EOT PET—no. (%)	
0–3	48 (87%)
>3	7 (13%)

**Table 2:** Contingency of interim PET2 results vs. end-of-treatment PET outcome.

PET2 Result	EOT PET Positive (Residual Disease)	EOT PET Negative (Remission)	Total Patients
PET2-positive (Deauville 4–5)	3 (true positive)	1 (false positive)	4
PET2-negative (Deauville 1–3)	4 (false negative)	47 (true negative)	51
Total	7	48	55

## Data Availability

The data that support the findings of this study are available from the corresponding author upon request.
